# Biophysical Behavior of Very High-Power Short-Duration Radiofrequency Ablation in Pulmonary Vein Isolation: Fast but Gently—Implications for a Successful Procedure

**DOI:** 10.3390/jcm12237332

**Published:** 2023-11-26

**Authors:** Eduardo Celentano, Ernesto Cristiano, Barbara Ignatiuk, Elena Bia, Lorenzo Girotto, Nicola Tarantino, Natasja M. S. De Groot

**Affiliations:** 1 Department of Electrophysiology, Humanitas Gavazzeni, Via Mauro Gavazzeni 21, 24125 Bergamo, Italy; ernesto.cristiano@gavazzeni.it (E.C.); barbara.ignatiuk@gavazzeni.it (B.I.); elena.bia@gavazzeni.it (E.B.); 2 Unit Translational Electrophysiology, Department of Cardiology, Lowlands Institute for Bioelectric Medicine, Erasmus University Medical Center, 3015 GD Rotterdam, The Netherlands; n.m.s.degroot@erasmusmc.nl; 3 Biosense Webster, Inc., Irvine, CA 91765, USA; lorenzo.girotto@yahoo.com; 4 Montefiore Medical Center, Cardiology Division, New York, NY 10467, USA; nicolatarantinomd@gmail.com

**Keywords:** ablation, atrial fibrillation, high power short duration, procedure, pulmonary vein isolation

## Abstract

The very high-power short-duration (vHP-SD) ablation strategy is an alternative for pulmonary vein isolation (PVI) in patients with paroxysmal atrial fibrillation (PAF). However, the acute procedural biophysical behavior of successful lesion creation by means of this technique is still unexplored. We performed a retrospective case–control study aimed at evaluating the behavior of vHP-SD ablation parameters with the QDOT MICRO™ ablation catheter (Biosense Webster) compared with standard radiofrequency (RF) ablation with the THERMOCOOL SMARTTOUCH^®^ ablation catheter. Twenty consecutive cases of symptomatic PAF treated with the QDOT MICRO™ ablation catheter from December 2022 to March 2023 were compared with cases treated with the standard technique. The acute procedural success of PVI was obtained in all cases with 2192 RF applications, and no adverse events occurred. Compared with the controls, vHP-SD cases featured a significant reduction in procedural time (47 ± 10 vs. 56 ± 12 min, *p* = 0.023), total RF time (3.8 [CI 3.4–4.6] vs. 21.2 [CI 18.4–24.9] min, *p* < 0.001), ablation phase time (25 ± 5 vs. 39 ± 9 min, *p* < 0.001), and irrigation volume (165 [CI 139–185] vs. 404 [CI 336–472] ml, *p* < 0.001). In vHP-SD RF ablation, a contact force of 5 g minimum throughout the 4 s of RF application appeared to be statistically significant in terms of an impedance drop of at least 10 Ohm (OR 2.63 [CI 1.37; 5.07], *p* = 0.003). In contrast, in the control group, the impedance drop depended linearly on the contact force. This suggests a different biophysical behavior of vHP-SD ablation. A maximum temperature and minimum contact force of >5 g independently predicted an effective impedance drop in vHP-SD. Increasing the contact force over 5 g during 4 s of vHP RF application might not be necessary to achieve a successful lesion.

## 1. Introduction

Paroxysmal atrial fibrillation (PAF) is a common cardiac arrhythmia associated with a fivefold increased risk of stroke [1]. Radiofrequency (RF) ablation of pulmonary veins (pulmonary vein isolation, PVI) is the current gold standard in the treatment of symptomatic PAF [2,3]. Several studies have shown that PVI is highly effective at restoring sinus rhythm and reducing AF symptoms [4]. Furthermore, PVI has been associated with a reduced risk of stroke, improved quality of life, and decreased health care utilization compared with traditional pharmacological therapies [4,5].

Classical strategies of PVI ablation consist of contact force catheters (CFs) creating point-by-point lesions at low-to-moderate power (20–40 W), with each one being applied for approximately 40–50 s [6] with an acceptable contact force (5–20 g). Because of the relatively long contact time between the catheter and the endocardium that is required to create a transmural lesion, stability in a contractile chamber is often inadequate [7,8], and collateral damage due to long RF delivery (i.e., atrioesophageal fistula) are a realistic concern.

High-power short-duration (HP-SD) ablation is an alternative technique that was conceived to guarantee effective lesion durability while sparing other organs from untoward injury by utilizing a higher power setting for a shorter duration of time compared with traditional RF [9]. This approach has been shown to be effective at achieving durable PVI and reducing AF recurrence rates [10,11]. Additionally, HP-SD ablation has been associated with shorter procedure times and shorter hospitalizations, maintaining safety requirements [10,11]. These results suggest that HP-SD ablation is a viable alternative to traditional RF techniques for the treatment of AF. The HP-SD approach does not exceed 70 W [12], while vHP-SD employs a catheter that is able to deliver as much as 90 W. However, procedural predictors of adequate lesion creation in vHP-SD ablation have not been investigated so far. 

The aim of this study was to assess the biophysical characteristics of vHP-SD PAF ablation parameters in generating effective lesions compared with standard RF ablation using the THERMOCOOL SMARTTOUCH^®^(Biosense Webster, Irvine, CA, USA) ablation catheter. This evaluation aims to assist clinicians by providing technical guidance to electrophysiologists, enabling the discrimination of simple, rapid, yet effective predictive values even before the application of energy.

## 2. Materials and Methods

### 2.1. Study Design

The present study is a single-center, non-randomized case–control analysis. Twenty consecutive patients with symptomatic paroxysmal AF (vHP-SD group) treated at our institution with the irrigated catheter QDOT MICRO™ (Biosense Webster, Irvine, CA, USA) from December 2022 to March 2023 were compared with an equivalent number of cases from a historical cohort who received PVI using the THERMOCOOL SMARTTOUCH^®^ ablation catheter (Biosense Webster, CA, USA) from May to November 2022 (a total of 40 cases). The study was approved by the local IRB and was in line with the ethical principles of the Declaration of Helsinki.

### 2.2. Study Population

Eligible participants were 18 years of age or older who were diagnosed with symptomatic paroxysmal AF with an electrocardiographically documented episode within 6 months before enrollment. They had no response or intolerance to at least 1 class I or III antiarrhythmic drug (AAD) and were able to comply with follow-up clinic visits. The exclusion criteria included the following: (1) previous surgical or catheter ablation for AF; (2) previous diagnosis of persistent or long-standing-persistent AF, coronary artery bypass graft, carotid stenting, or endarterectomy within the past 6 months; (3) left atrial thrombus documented at preprocedural images; (4) left ventricle ejection fraction < 40%; (5) left atrial diameter > 50 mm; (6) thromboembolic event within the past 12 months; and (7) uncontrolled New York Heart Association functional class III or IV heart failure.

### 2.3. Procedural Protocol

**Figure 1:**
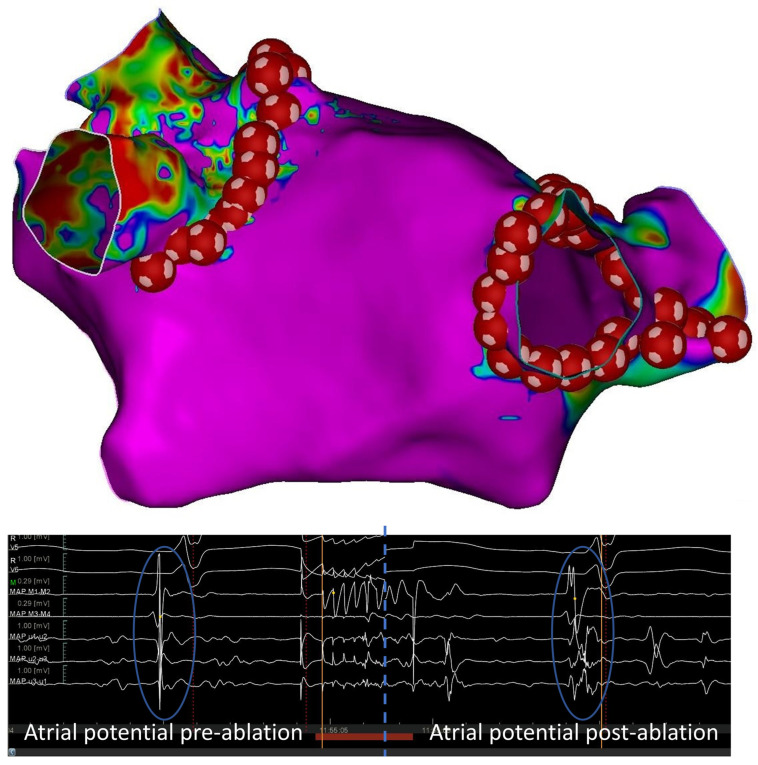
Voltage map of the left atrium. Both pulmonary veins were isolated by a point-by-point vHP-SD ablation (represented by red tags). Double atrial potentials were obtained after an application of 90 W for 4 s.

All procedures were performed under general anesthesia and after transseptal puncture; electroanatomic mapping was performed with the CARTO 3 system using a PENTARAY catheter (Biosense Webster, Inc., Irvine, CA, USA). The ablation strategy consisted of point-by-point, wide-area, circumferential PVI ablation (Figure 1).

All linear lesions required confirmation of a bidirectional conduction block by mapping maneuvers and/or pacing. A 20-minute waiting period was required post-ablation before pacing procedures and/or infusion of cardiac medications to induce AF or reconnection (e.g., isoproterenol 2 to 20 mg/min, adenosine). 

### 2.4. The vHP-SD Group

In the vHP-SD group, a QDOT MICRO™ (D-F curve) ablation catheter was used in the QMODE+ (90 W, 4 s) mode for all applications. The target for the temperature-controlled ablation was 55 °C (cutoff at 60 °C) based on the hottest surface thermocouple. For the anterior wall of the left atrium, the interlesion distance (calculated from the center of an ablation tag to the center of the adjacent one) was ≤4 mm; otherwise, it was ≤6 mm. In the QMODE+ mode, the power and irrigation are adjusted based on the target temperature. Two seconds prior to RF delivery, the flow rate rises from 2 to 8 mL/min to cool down the tissue, and 90 W of RF is applied for 4 s. If the target temperature is reached during ablation, the flow rate increases to keep the temperature below the target. Conversely, if the temperature continues to rise despite the action of irrigation, then the power starts to decrease to maintain the target. After creating a single lesion, the flow rate remains at 8 mL/min for an additional 4 s before decreasing back to 2 mL/min.

### 2.5. Control Group

In the control group, a THERMOCOOL SMARTTOUCH^®^ (D-F curve) catheter was used in the power-controlled mode. For the posterior wall, the power was set to 25 W with a target AI of 360; otherwise, the power was set to 30 W with a target AI of 430. The minimum interlesion distance was maintained at ≤6 mm in the entire chamber.

### 2.6. Statistical Methods

In the experimental and control groups, the following parameters were analyzed for each RF application: power; duration; minimum and maximum temperature; baseline and minimum impedance; and minimum, maximum, and mean contact force. Additionally, the overall RF duration of the mapping phase and ablation phase (time between the first and last RF application), LA dwelling time, and irrigation volume were compared.

Statistical analysis was carried out using R software (version 4.2.2). For all tests, a *p*-value of <0.05 (two-tailed) was considered statistically significant. Descriptive data are presented as proportions or means ± standard deviations (SDs) for continuous variables with a normal distribution and as medians and interquartile ranges for nonparametric variables, as appropriate. Differences between groups were assessed by using Student’s *t*-test or the Mann–Whitney U test accordingly. The normal distribution of data was assessed using indirect methods, including the kurtosis and symmetric indices and with the Shapiro–Wilk test or Kolmogorov–Smirnov test as appropriate for the degrees of freedom. Pearson’s χ2 test, Fisher’s exact test, or continuity correction were used to compare categorical variables. Univariate and multivariate analyses were obtained using a linear mixed effect model, where patients represent the random parameter while ablation data represent the fixed parameters. The best predictive model was chosen using Akaike information criterion (AIC). The best cutoff value for an impedance drop of >10 Ohm was calculated using the receiver operator curve (ROC) and Youden’s J index. The final predictive model of an impedance drop of >10 Ohm was obtained using a multivariable logistic mixed effect model.

## 3. Results

### 3.1. Baseline Data

Among 40 patients, 21 (52.5%) were male, and the average age was 63.2 ± 8.5 years. The mean left ventricular ejection fraction (LVEF) was 59.9% ± 5.0. Seven patients (17.5%) presented hypertension, which prevailed equally between the groups. No echocardiographic anomaly was present; in particular, no ventricular dysfunction or atrial enlargement was observed in either group, and no significant differences were present in the clinical data between the two groups (Table 1).

### 3.2. Procedural Characteristics

At the end of the procedure, all pulmonary veins were successfully isolated in both groups, and no adverse events occurred. A total of 2192 RF applications were collected, 1223 from the vHP-SD group and 969 from the control group, with an average number of applications per patient of 61 [59; 64] and 48 [46; 54], respectively (*p* = 0.002). A significant reduction in left atrial (LA) dwelling time was observed (47 ± 10 min vs. 56 ± 12 min, *p* = 0.023), associated with a significant reduction in total RF time (3.8 [3.4; 4.6] min vs. 21.2 [18.4; 24.9] min, *p* < 0.001); meanwhile, no difference in mapping time was noted (14 ± 3.5 min vs. 13 ± 3.3 min, *p* = 0.278). Instead, when considering the ablation phase as the time between the first and the last RF application, we found for the vHP-SD group a mean value of 25 ± 5 min vs. 39 ± 9 min in the control group (*p* < 0.001), which corresponds to a 36% decrease in the vHP-SD group (Table 2).

Moreover, the mean irrigation fluid volume of the ablation catheter during the entire procedure was 164.6 mL in the vHP-SD group and 403.7 mL in the control group (*p* < 0.001), resulting in a fluid reduction of 59%.

### 3.3. Biophysical Behavior of Ablation Parameters in the vHP-SD Group vs. Controls

The mean contact force during RF with the QDOT MICRO™ ablation catheter was 9.1 ± 3.8 g, and with the THERMOCOOL SMARTTOUCH^®^ catheter, it was 9.2 ± 3.6 g (*p* = 0.63).

**Figure 2:**
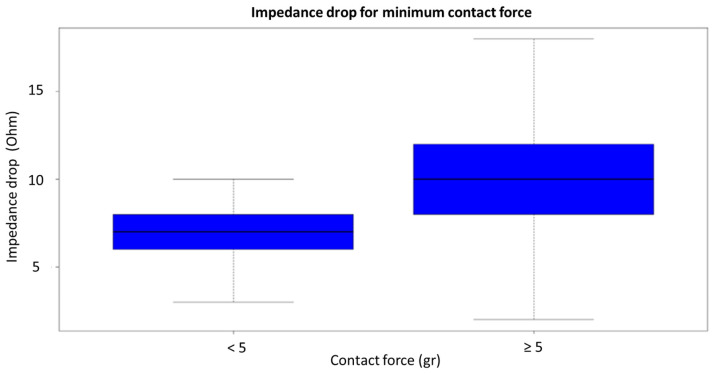
The graph shows an impedance drop of larger than 10 ohms when the contact force is less or more than 5 g during vHP-SD ablation.

In the univariate analysis, in the vHP-SD group, the maximum temperature and minimum force of contact registered during the single RF application correlated with an impedance drop of >10 ohms, which has been found to be the best cutoff for the production of an effective lesion [13] (Figure 2).

Using the univariate parameters, we obtained linear mixed effect models corrected by the starting impedance, and the best model was chosen by the AIC value. The model showed a significant linear dependence between the impedance drop and the temperature increase obtained during RF application in both strategies (0.5 ± 0.03, *p* < 0.001). Meanwhile, the mean contact force was only linearly predictive for the impedance drop in the control group (3.63 ± 0.53, *p* < 0.001) (Table 3). In the ROC analysis, a contact force of 5 g throughout the whole RF application had modest but statistically significant sensitivity and specificity after bootstrapping (AUC 0.60 [0.57–0.63], accuracy 0.57 [0.53–0.61], sensitivity 0.63 [0.59–0.66], specificity 0.52 [0.50–0.56]). When a minimum contact force was added as a binary value (≥5 g or <5 g), the value became a significant predictor of the impedance drop in the vHP-SD group (for vHP-SD 1.9 ± 0.84, *p* = 0.024; for control, 3.5 ± 1.7, *p* = 0.036). Finally, the multivariable logistic mixed effect model confirmed that the cutoff of 5 g for the minimum contact force was a strong predictor of an impedance drop of >10 Ohm in the vHP-SD group (OR 2.63 [1.37; 5.07], *p* = 0.003), but not in the control group (OR 0.69 [0.28; 1.72], *p* = 0.419).

## 4. Discussion

The study confirms the previously described advantages of vSHP-SD in terms of safety.

In this study, the total RF time was statistically and numerically lower in the vHP-SD group than in the control group, as was the ablation phase, despite a higher number of RF applications; this is explained by the shorter interlesion distance kept in the anterior wall. This is in agreement with the literature and provides additional evidence supporting the concept that by reducing the RF time and catheter dwelling time, the theoretical risk of atrial wall or esophageal lesion [14,15] also decreases.

Moreover, there was a 59% reduction in total irrigation volume in the vHP-SD group, which can be useful in patients with congestive heart failure, where fluid overload should be as low as possible. Overall, the time reduction and possible periprocedural risk reduction can impact the total hospitalization time and costs. Nonetheless, further investigation with a larger population is warranted.

This is the first report on a comparison of the biophysical behavior of ablation parameters between vHP-SD and conventional ablation modalities (QMode vs. Thermocool).

We collected and analyzed the power, temperature, CF, time, and impedance for all RF applications. Our data indicate that an impedance drop of more than 10 Ohm is suggestive of a transmural lesion.

**Figure 3:**
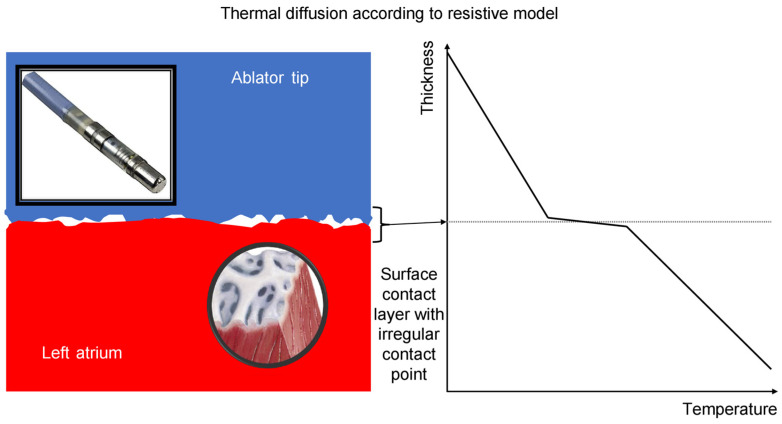
The figure illustrates the thermal diffusion between two layers. In this physical model, when there is imperfect contact between the layers, heat diffusion is slowed down due to the presence of “blood pockets.” In vHP-SD ablation, once the minimum contact force required for optimal surface matching (>5 g) is achieved, increasing the contact force does not lead to increased heat diffusion within a fixed time period (4 s).

Lesion size correlates with contact force and contact time, and validation in animal models has shown a linear correlation between adequate contact and lesion transmurality, despite constant power and identical peak contact forces [16]. As contact force is an important factor in lesion creation, increased catheter–tissue contact has been associated with a larger impedance decrease during RF ablation [16,17,18], and in the absence of sufficient catheter–tissue contact, an impedance decrease of 10 ohms cannot be achieved. According to our findings in the control group, an increase in the mean contact force corresponded linearly to an impedance drop; meanwhile, in the vHP-SD ablation group, due to a different ablation approach, only a minimum contact force of >5 g during the 4 s of RF application was the main condition for achieving an effective impedance drop. This might be explained by considering the physical model (Figure 3). The standard RF ablation technique (low-to-moderate power) creates lesions as a result of resistive (endocardial) and conductive (transmural) myocardial heating; in contrast, with a vHP-SD strategy, the lesion is created almost exclusively by resistive heating [10,19]. More specifically, due to biophysical limitations, the interface contact between two solid surfaces such as an irrigating catheter tip and a contractile atrial wall is imperfect, as continuously variable fluid pockets between the two surfaces create an uneven and unstable juncture (Figure 2). Therefore, once the best surface matching is obtained with a stable position of the QDOT MICRO™ ablation catheter and a minimum contact force necessary to minimize the micro fluid-pockets is achieved, the RF application should be effective [20]. To enhance clarity, it is worth noting that imperfect contact between two layers hinders resistive heat diffusion on the contact surface. This slowdown is attributed to the existence of “blood pockets,” which exhibit distinct heat diffusion characteristics. In vHP-SD ablation, reaching the minimum contact force necessary for optimal surface matching (in our analysis, at least 5 g) is crucial. Importantly, our findings indicate that beyond this point, further increases in contact force do not result in augmented resistive heat diffusion within a fixed time period (4 s).

Turning our focus back to clinical and practical implications, Yamashita [21] compared HP with a low-power setting in the ablation of AF using the ﻿RHYTHMIA HDxTM Mapping System, Boston Scientific, and an open-irrigated ablation catheter with mini-electrodes (IntellaNav MIFI OI), using local impedance (LI) as a target. They observed a larger impedance change and shorter time to minimum LI with the HP setting, concluding that it may result in more transmural lesions. Although both studies were looking at the minimal effective impedance drop, the technologies, tools, and methodologies differ substantially and cannot be directly comparable. The Carto system uses a generator impedance; the Rhythmia system does not have a vHP option; and in the Yamashita study, CF was not available. Still, this interesting study confirms that impedance is an attractive parameter to monitor during ablation procedures.

Another pertinent consideration pertains to the transmurality of lesions and their clinical implications. Yamashita [21] provided indirect evidence of transmural lesions during HP-SD, relying solely on acute unipolar electrogram analysis. However, these data in acute settings may be influenced by local edema and do not confirm a lasting transmural lesion. In our protocol, the thorough electrical isolation was verified through bidirectional electrical isolation of antral PV tissue, supporting the establishment of a complete “electrical transmural lesion.” Nevertheless, definitive confirmation of permanent transmural lesions can only be achieved in vivo through MRI.

The positive implications of resistive heat diffusion are noteworthy; a decreased energy diffusion through the wall can mitigate atrial tissue damage, lowering the risk of perforation, significant scar reactions, PV stenosis, and extracardiac tissue damage such as the perilous atrioesophageal fistula. On the flip side, incomplete transmural lesions may compromise ablation effectiveness, especially in certain subgroups like long-standing persistent AF. De Groot et al. [22,23] demonstrated in this subgroup that in permanent and long-standing persistent AF, fibrillation waves stem from epicardial breakthroughs originating in deeper atrial wall layers. It is important to note that our study exclusively focused on paroxysmal AF patients. Further research is imperative to assess the efficacy of vHP-SD in other AF subgroups.

The present investigation is a single-center non-randomized endeavor with a small population size; however, our findings can potentially be confirmed in larger cohorts.

## 5. Conclusions

In summary, the key findings from our observational study are as follows:

1. Procedural characteristics:
  - vHP-SD ablation demonstrated reduced RF time, ablation phase time, LA dwelling time, and irrigation volume compared with the standard technique. These findings have the potential to lower the risk associated with transmural lesions and fluid overload. This suggests the feasibility of achieving fewer complications while maintaining acute success, though further studies with larger populations are essential for confirmation.
2. Physical behavior:
  - In both arms, temperature increase proved to be predictive of an impedance drop of >10 Ohm and, consequently, tissue lesion.
  - In vHP-SD ablation, a minimum contact force of >5 g (rather than the mean contact force throughout the application) predicted an impedance drop of >10 Ohm and tissue lesion in our population. This differs from the standard technique, where the mean contact force (not the minimum value) is predictive of an impedance drop.

In conclusion, surpassing a contact force of 5 g during a 4-s vHP RF application may not be necessary for achieving an effective lesion. This underscores one of the primary advantages of the new technique, highlighting that stability alone suffices for an effective lesion.

## Figures and Tables

**Table 1:** Baseline clinical, therapeutic, and echocardiographic characteristics of patients.

Clinical Parameters		Control (20)	vHP-SD (20)	*p*-Value
Male, n (%)	21 (52.5)	13 (65.5)	8 (40)	0.123
Age (years)	63.2 ± 8.5	64.4 ± 7.7	61.5 ± 9.7	0.383
BMI (kg/m^2^)	26.3 ± 2.9	26.9 ± 3.0	25.5 ± 2.7	0.223
Hypertension, n (%)	7 (17.5)	3 (15)	4 (20)	0.677
CAD, n (%)	0 (0)	0 (0)	0 (0)	0.999
Diabetes, n (%)	6 (15)	4 (20)	2 (10)	0.376
Smokers, n (%)	7 (17.5)	3 (15)	4 (20)	0.677
Previous ablation, n (%)	0 (0)	0 (0)	0 (0)	0.999
CHA2DS2VASC score	1.2	1.1 ± 0.7	1.3 ± 0.6	0.338
Time from first episode to ablation (mo)	37 ± 19	40 ± 20	35 ± 17	0.399
**Medications**				
Ic AAD, n (%)	9 (22.5)	4 (20)	5 (25)	0.705
Amiodarone, n (%)	4 (10)	2 (10)	2 (10)	0.999
Anticoagulant, n (%)	12 (30)	5 (25)	7 (35)	0.490
Betablocker, n (%)	15 (37.5)	6 (30)	9 (45)	0.327
RAAS, n (%)	6 (15)	3 (15)	3 (15)	0.999
Other, n (%)	3 (7.5)	2 (10)	1 (5)	0.520
**Echocardiography**				
LV-EF (%)	59.9 ± 5.0	60.5 ± 4.5	59.3 ± 5.7	0.626
LA diameter, mm	37 ± 8	36.1 ± 10	38.3 ± 6	0.404
LA volume (mL/mq)	30 ± 6	29 ± 5	31 ± 7	0.305
TAPSE, mm	20 ± 5	20 ± 6	21 ± 4	0.539
PASP, mmHg	28 ± 6	27 ± 7	29 ± 5	0.305

Data are expressed as a number and the percentage is in parentheses for nominal data; plus-minus values are the means ± standard deviation. BMI, body mass index; CAD, coronary artery disease; Ic AAD, antiarrhythmic drugs of class Ic based on the Vaughan Williams (VW) classification; RAAS renin angiotensin aldosterone system; LV-EF, left ventricle ejection fraction; LA, left atrium; TAPSE, tricuspid annular plane excursion; PASP pulmonary artery systolic pressure.

**Table 2:** Procedural characteristics between the two groups.

	vHP-SD	Control	*p*-Value
Total RF time (s)	229 [202; 273]	1270 [1106; 1492]	<0.001
RF applications (n)	58 [51; 64]	46 [41; 55]	0.002
Irrigation fluid (mL)	165 [139; 185]	404 [336; 472]	<0.001
Mapping time (min)	14 ± 3.5	12.5 ± 3.3	0.278
Ablation phase (min)	25 ± 5	39 ± 9	<0.001
LA dwelling time (min)	47 ± 10	56 ± 12	0.023

Data are expressed as the median with the interquartile range in square brackets for non-normally distributed data; plus-minus values are the means ± standard deviation. RF, radiofrequency.

**Table 3:** Linear mixed effect models using different contact force variables and binary values for the predictive parameter of the impedance drop. In the last column, the logistic mixed effect model for the predictive parameter of an impedance drop of >10 Ohm after obtaining the best cutoff value by AUC analysis.

	Model 1	*p*-Value	Model 2	*p*-Value	Model 3	*p*-Value	Model 4	*p*-Value	Log Model	*p*-Value
T increase for ST	0.36 [0.28; 0.44]	<0.001	0.55 [0.45; 0.65]	<0.001	0.55 [0.47; 0.63]	<0.001	0.74 [0.64; 0.84]	<0.001	1.34 [1.25; 1.43]	<0.001
T increase for QDot	0.40 [0.28; 0.52]	<0.001	0.52 [0.36; 0.68]	<0.001	0.52 [0.38; 0.66]	<0.001	0.36 [0.28; 0.44]	<0.001	1.24 [1.19; 1.31]	<0.001
Starting impedance	0.39 [0.35; 0.43]	<0.001	0.13 [0.11; 0.15]	<0.001	0.14 [0.12; 0.16]	<0.001	0.39 [0.35; 0.43]	<0.001	1.12 [1.02; 1.04]	<0.001
F min for ST	-	-	-	-	0.75 [0.41; 1.09]	<0.001	-	-	-	-
F med for ST	-	-	2.41 [1.45; 3.37]	<0.001	-	-	-	-	-	-
F max for ST	2.75 [2.09; 3.41]	<0.001	-	-	-	-	-	-	-	-
F min for QDot	-	-	-	-	−0.26 [−0.62; 0.10]	0.171	-	-	-	-
F med for QDot	-	-	−0.31 [−1.11; 0.49]	0.440	-	-	-	-	-	-
F max for QDot	0.04 [−0.58; 0.66]	0.910	-	-	-	-	-	-	-	-
F min > 5 g for Qdot	-	-	-	-	-	-	2.83 [1.17; 4.49]	0.005	2.63 [1.37; 5.07]	0.003
F min > 5 g for ST	-	-	-	-	-	-	1.43 [−1.51; 4.37]	0.092	0.69 [0.28; 1.72]	0.419

Linear models were obtained between the impedance drop and various predictive parameters. Numbers represent the coefficients of the linear model; in square brackets, confidence intervals (CIs) at 95%. Model 1: linear model with maximum contact force; Model 2: linear model with average contact force; Model 3: linear model with minimum contact force; Model 4: linear model with cutoff < or > 5 g contact force. Log model: logistic model for the predictive parameter of an impedance drop of >10 Ohm. Numbers represent odd ratios (ORs); in square brackets CI at 95%. QDot, QDOT mode ablation technology; ST, SMARTTHOUCH (control group); T, temperature; g, grams; F, force.

## Data Availability

The data that support the findings of this study are available from the corresponding author upon reasonable request.
